# Supplementary material for: Ciliary neuropeptidergic signaling dynamically regulates excitatory synapses in postnatal neocortical pyramidal neurons
Source: eLife. 2021 Mar 2;10:e65427. doi: 10.7554/eLife.65427 (PMC7952091; doi:10.7554/eLife.65427)
Supplement: Supplementary file 1. [file elife-65427-supp1.docx]

**Supplementary File 1.** Statistical analyses used.

| **Figure** | **Sample size No. of experiments** | **Statistical test** | **P-value** |
| --- | --- | --- | --- |
| 1C | Excitatory= 46,  inhibitory =44  5 dissociations | Wilcoxon rank-sum | Excitatory vs inhibitory p=0.35 |
| 1E,F | Control = 34  shArl13b_1 = 43 shArl13b_2 = 40  4 dissociations | Kruskal-Wallis with Dunn’s multiple comparisons test | E:  Control vs shArl13b_1 p<0.001  Control vs shArl13b_2 p<0.001  F:  Control vs shArl13b_1 p<0.001  Control vs shArl13b_2 p<0.001 |
| 1 H,I | Control = 32  shArl13b_1 = 28    4 dissociations | Wilcoxon rank-sum test | H:  Control vs shArl13b_1 p=0.52  I:  Control vs shArl13b_1 p=0.29 |
| 2C,D | (24h)  Control = 25  shArl13b_1 = 24  (48h)  Control = 32  shArl13b_2 = 19 shIft88/shCep164 = 39  4 dissociations | LMM with Dunnett-type correction for multiple comparisons | C:  (24h)  Control vs shArl13b_1 p<0.001  (48h)  Control vs shArl13b_2 p=0.001  Control vs shIft88/shCep164 p=0.001  D:  (24h)  Control vs shArl13b_1 p=0.07  (48h)  Control vs shArl13b_2 p=0.011  Control vs shIft88/shCep164 p=0.016 |
| 2F,G | (24h)  Control = 17  shArl13b_1 = 22  4 dissociations  (48h)  Control = 25  shArl13b_2 = 19 shIft88/shCep164 = 23  3 dissociations | LMM with Dunnett-type correction for multiple comparisons | F:  (24h)  Control vs shArl13b_1 p=0.25  (48h)  Control vs shArl13b_2 p=0.50  Control vs shIft88/shCep164 p=0.64  G:  (24h)  Control vs shArl13b_1 p=0.86  (48h)  Control vs shArl13b_2 p=0.57  Control vs shIft88/shCep164 p=0.98 |
| 3A,D,E | Control = 24  shArl13b_1 = 24 shArl13b_2 = 23  >5 dissociations | Kruskal-Wallis with Dunn’s multiple comparisons test | A:  Control vs shArl13b_1 p= 0.007  Control vs shArl13b_2 p=0.007  D:  Control vs shArl13b_1 p=0.080  Control vs shArl13b_2 p=0.71  E:  Control vs shArl13b_1 p=1  Control vs shArl13b_2 p=0.097 |
| 3B,C | Control = 24  shArl13b_1 = 24 shArl13b_2 = 23  >5 dissociations | Kruskal-Wallis with Bonferroni correction | B:  Control vs shArl13b_1 p<0.001  Control vs shArl13b_2 p<0.001  C:  Control vs shArl13b_1 p=1  Control vs shArl13b_2 p=0.25 |
| 4A | Control = 32  shArl13b_1 = 25  >5 dissociations | Wilcoxon rank-sum test | Control vs shArl13b_1  p=0.032 |
| 4B | Control =18  shArl13b_1 = 20  3 dissociations | ANOVA for repeated measures | Control vs shArl13b_1:  30 pA p=1.00  50 pA p=0.99  100 pA p=0.82  200 pA p=0.84  300 pA p=0.88  400 pA p=0.99 |
| 5B | n=150 neurons per layer  3 animals. |  |  |
| 5D | n=  GAD67+ = 115  ChAT+ = 31,  PV+ = 100,  SOM+ = 110,  VIP+=150  3 animals. |  |  |
| 5F | n = 515 neurons  4 dissociations. |  |  |
| 6B,C | (6h)  Control = 23  L-796,778 = 10  MK-4256 = 17  (18h)  Control = 35,  L-796,778 = 33  MK-4256 = 17  (24h)  Control = 87  L-796,778 = 17  MK-4256 =40  ≥3 dissociations | LMM with Dunnett-type correction for multiple comparisons | B:  (6h)  Control vs L-796-778 p=0.96  Control vs MK-4256 p=1.00  (18h)  Control vs L-796-778 p=0.78  Control vs MK-4256 p= 0.073  (24h)  Control vs L-796-778 p= 0.003  Control vs MK-4256 p< 0.001  C:  (6h )  Control vs L-796-778 p=0.76  Control vs MK-4256 p=0.60  (18h)  Control vs L-796-778 p=0.02  Control vs MK-4256 p=1.00  (24h)  Control vs L-796-778 p= 0.02  Control vs MK-4256 p= 0.28 |
| 6E | GFP control = 35  GFP + L-796,778 = 46  shIft88/shCep164/GFP = 36  shIft88/shCep164/GFP + L-796,778 = 31  3 dissociations | Kruskal-Wallis with Dunn’s multiple comparisons test | Control GFP vs GFP + L-796-778 p= 0.01  Control GFP vs shIft88/shCep164 p= 0.01  Control GFP vs shIft88/shCep164 + L-796-778 p= 0.0022  GFP + L-796-778 vs shIft88/shCep164 p<0.0001  GFP + L-796-778 vs shIft88/shCep164 + L-796-778 p<0.0001  shIft88/shCep164 vs shIft88/shCep164 + L-796-778 p=0.45 |
| Figure 1 – Supp.1A,B | Control = 19  shArl13b_2 = 26  3 dissociations | Wilcoxon rank-sum test | A: Control vs shArl13b_2 p=<0.0001  B: Control vs shArl13b_2 p=0.0048 |
| Figure 1 – Supp.1D,E | Control = 21  shArl13b_2 = 14 shIft88/shCep164 =17  3 dissociations | Kruskal-Wallis with Dunn’s multiple comparisons test | D:  Control vs shArl13b_2 p=0.74  Control vs shIft88/shCep164 p=0.82  E:  Control vs shArl13b_2 p=0.52  Control vs shIft88/shCep164 p=0.13 |
| Figure 1 – Supp.1F,G | (24h)  Control = 23  shIft88/shCep164 = 25  3 dissociations  (48h)  Control = 43,  shCep164/GFP = 20,  shIft88/GFP = 17,  shIft88/shCep164/GFP = 23  3 dissociations. | (24h)  Wilcoxon rank-sum test  (48h)  Kruskal-Wallis with Dunn’s multiple comparisons test | F:  (24h)  Control vs shIft88/shCep164 p=0.033  (48h)  Control vs shCep164/GFP p= 0.16  Control vs shIft88/GFP p= 0.0035  Control vs shIft88/shCep164/GFP p= <0.0001  shCep164/GFP vs shIft88/GFP p= 0.14  shIft88/GFP vs shIft88/shCep164/GFP p= 0.032  shCep164/GFP vs shIft88/shCep164/GFP p= 0.00018  G:  (24h)  Control vs shIft88/shCep164 p=0.24  (48h)  Control vs shCep164/GFP p= 0.29  Control vs shIft88/GFP p= 0.017  Control vs shIft88/shCep164/GFP p= <0.0001  shCep164/GFP vs shIft88/GFP p= 0.19  shIft88/GFP vs shIft88/shCep164/GFP p= 0.024  shCep164/GFP vs shIft88/shCep164/GFP p= 0.00016 |
| Figure 2 – Supp.1A | (24h)  Control = 25  shArl13b_1 = 24  (48h)  Control = 32  shArl13b_2 = 19 shIft88/shCep164 = 39  4 dissociations | LMM with Dunnett-type correction for multiple comparisons | (24h)  Control vs shArl13b_1 p=0.017  (48h)  Control vs shArl13b_2 p=0.14  Control vs shIft88/shCep164 p=0.04 |
| Figure 2 – Supp.1B | (24h)  Control = 17  shArl13b_1 = 22  4 dissociations  (48h)  Control = 25  shArl13b_2 = 19 shIft88/shCep164 = 23  3 dissociations | LMM with Dunnett-type correction for multiple comparisons | (24h)  Control vs shArl13b_1 p=0.99  (48h)  Control vs shArl13b_2 p=0.99  Control vs shIft88/shCep164 p=0.96 |
| Figure 5 – Supp.1B | NeuN+ = 1,520  GAD67+ = 253  6 animals |  |  |
| Figure 6 – Supp.1A | (6h)  Control = 23  L-796,778 = 10  MK-4256 = 17  (18h)  Control = 35  L-796,778 = 33  MK-4256 = 17  (24h)  Control = 87  L-796,778 = 17  MK-4256 =40  ≥3 dissociations | LMM with Dunnett-type correction for multiple comparisons | (6h)  Control vs L-796-778 p=0.96  Control vs MK-4256 p=0.99  (18h)  Control vs L-796-778 p=0.22  Control vs MK-4256 p=0.29  (24h)  Control vs L-796-778 p=0.04  Control vs MK-4256p=0.002 |
| Figure 6 – Supp.1B,C | Control = 87  0.5 μM L-796,778 = 26  1 μM L-796,778 = 25  2 μM L-796,778 = 17  0.125 μM MK-4256 = 19  0.5 μM MK-4256 = 15  1 μM MK-4256 = 40  ≥3 dissociations. | LMM with Dunnett-type correction for multiple comparisons | B:  Shank3 intensity:  Control vs 0.5 μM L-796,778 p =0.96  Control vs 1 μM L-796,778 p =0.78  Control vs 2 μM L-796,778 p =0.004  Control vs 0.125 μM MK-4256 p =0.37  Control vs 0.5 μM MK-4256 p =0.37  Control vs 1 μM MK-4256 p =0.001  VGlut1 intensity:  Control vs 0.5 μM L-796,778 p =0.19  Control vs 1 μM L-796,778 p =0.47  Control vs 2 μM L-796,778 p = 0.037  Control vs 0.125 μM MK-4256 p=0.40  Control vs 0.5 μM MK-4256 p=0.99  Control vs 1 μM MK-4256 p =0.002  C:  Density  Control vs 0.5 μM L-796,778 p=0.25  Control vs 1 μM L-796,778 p=0.98  Control vs 2 μM L-796,778 p=0.026  Control vs 0.125 μM MK-4256 p=0.42  Control vs 0.5 μM MK-4256 p=0.89  Control vs 1 μM MK-4256 p=0.28 |
| Figure 6 – Supp.1D | Control = 63  2 μM L-796,778 = 7  1 μM MK-4256 = 33  ≥3 dissociations | LMM with Dunnett-type correction for multiple comparisons | Control vs 2 μM L-796,778 p=0.21  Control vs 1 μM MK-4256 p=0.32 |
